# Prognostic Optical Coherence Tomography Biomarkers in Neovascular Age-Related Macular Degeneration

**DOI:** 10.3390/jcm12093049

**Published:** 2023-04-22

**Authors:** Baraa Nawash, Joshua Ong, Matthew Driban, Jonathan Hwang, Jeffrey Chen, Amrish Selvam, Sashwanthi Mohan, Jay Chhablani

**Affiliations:** 1 Department of Ophthalmology, University of Pittsburgh School of Medicine, Pittsburgh, PA 15213, USA; ban47@pitt.edu (B.N.);; 2 Michigan Medicine, University of Michigan, Ann Arbor, MI 48104, USA; 3 Ophthalmology, Medcare Hospital LLC, Dubai P.O. Box 215565, United Arab Emirates; 4 Education and Research, Rajan Eye Care Hospital Pvt Ltd., Chennai 600042, India

**Keywords:** neovascular age-related macular degeneration, optical coherence tomography, visual acuity, prognostic factors, anti-VEGF, future advancements

## Abstract

Optical coherence tomography has revolutionized the diagnosis and management of neovascular age-related macular degeneration. OCT-derived biomarkers have the potential to further guide therapeutic advancements with anti-vascular endothelial growth factor; however, the clinical convergence between these two tools remains suboptimal. Therefore, the aim of this review of literature was to examine the current data on OCT biomarkers and their prognostic value. Thirteen biomarkers were analyzed, and retinal fluid had the strongest-reported impact on clinical outcomes, including visual acuity, clinic visits, and anti-VEGF treatment regimens. In particular, intra-retinal fluid was shown to be associated with poor visual outcomes. Consistencies in the literature with regard to these OCT prognostic biomarkers can lead to patient-specific clinical decision making, such as early-initiated treatment and proactive monitoring. An integrated analysis of all OCT components in combination with new efforts toward automated analysis with artificial intelligence has the potential to further improve the role of OCT in nAMD therapy.

## 1. Introduction

Age-related macular degeneration (AMD) is one of the most common causes of severe, irreversible vision loss worldwide, predominantly affecting the elderly. With a continually aging population, it is estimated that the projected number of people afflicted with the disease will increase to 288 million by 2040 [1].

AMD occurs in two forms: neovascular “wet” AMD (nAMD) and non-neovascular “dry” AMD, or a combination of the two, all of which can result in devastating central vision loss. nAMD is characterized by choroidal neovascularization (CNV), and although it accounts for less than 20% of total AMD incidence, it is responsible for 90% of severe vision loss due to AMD [2,3]. nAMD negatively impacts the quality of life and therefore appropriate diagnosis and management is imperative.

Gold-standard treatment for nAMD includes intravitreal injections of anti-vascular endothelial growth factor (anti-VEGF), such as aflibercept, ranibizumab, and bevacizumab [4]. At the time of initial evaluation and treatment follow-up, imaging modalities are employed to detect the presence and activity of CNV. Spectral–domain optical coherence tomography (SD-OCT) has become the primary imaging tool in the management of AMD [3,5]. It provides anatomic information about retinal layers, retinal pigment epithelium (RPE), and the choroid, while creating cross-sectional, high-fidelity images of the macula and the optic nerve [3,5].

OCT has become a means to predict and evaluate treatment response, and a method to further guide treatment options [6]. Although fluorescein angiography (FA) can be regarded as the gold-standard imaging modality to diagnose neovascularization, OCT has progressively emerged as the first-line diagnostic tool [6]. There have been several limitations to FA which has led to a diagnostic paradigm shift in retinal imaging. Limitations of FA include invasiveness, side-effects, and allergic reactions [7]. 

The fast and non-invasive benefits of OCT have strengthened its application as a key instrument for decision-making. It has been reported that OCT images have the best diagnostic accuracy in monitoring nAMD disease states [8]. Data have shown that findings on OCT can indicate the onset or progression of nAMD before a patient reports appearance of new symptoms or even changes in visual acuity measurement [8]. Therefore, this highlights the importance of key OCT findings or biomarkers, as a component toward personalized anti-VEGF treatment, disease control, and reduced burden on monitoring [9].

The literature indicates several key OCT biomarkers associated with visual acuity and treatment outcomes. Biomarkers, or biological makers, are defined as medical signs that represent a diseased or non-diseased state [10]. Biomarkers are often implemented as endpoints in randomized control trials in nAMD research, and serve as a signal for the progression or regression of disease [10]. OCT biomarkers in the context of this study are image-based points in the development, pathogenesis, or progression of nAMD. 

As one of the earliest and most intuitive OCT markers to interpret, SD-OCT measured central retinal thickness (CRT) has been shown to have conflicting associations with the best-corrected visual acuity (BCVA) [9]. This may be attributed to several factors such as technical measurement differences, poor fixation and patient motion, and, importantly, CRT considers many retinal layers, which in themselves can influence the BCVA [9]. Therefore, additional biomarkers have been studied, such as the distribution of fluid present within the retina, subretinal, and intraretinal fluid [9]. These biomarkers may provide valuable insights into potential treatment responses.

The aim of this narrative review is to evaluate and examine the relationship between OCT imaging biomarkers and nAMD disease progression, treatment response, and the BCVA outcomes. This narrative review further aims to shed light on the OCT biomarkers linked to positive and negative prognostic indicators and their potential consequence in clinical management.

## 2. Materials and Methods

A comprehensive database search on OCT biomarkers was performed. Biomarkers in this study were defined as imaging indicators that detect change in the physiologic state of the patient’s disease onset or progression given a therapeutic [10]. The prognostic indications were focused on visual acuity, disease progression, and anti-VEGF injections. 

A structured search of the PubMed database was performed in January 2023, utilizing key phrases, words, and combinations, which include, but are not limited to: “AMD”, “nAMD”, “wet AMD”, “OCT Biomarkers”, “predictive factors in nAMD”, “prognostic indicators OCT” and/or “Imaging prognostic model nAMD” with the URL https://www.ncbi.nlm.nih.gov/pmc/ accessed 1st through 20th of January 2023. The search results were reviewed and identified as relevant based on the title. If the relevance was deemed unclear, the abstract and the manuscript were read in full prior to the inclusion. The references of each paper were further assessed for key information and to ensure the robust capture of all OCT imaging findings noted to be associated with nAMD outcomes. 

Articles included in the future directions were identified within the original data search, the reference section of relevant articles, or with the added search of the key phrase “future directions OCT imaging” in PubMed. The goal of this literature review, therefore, is a narrative description of OCT’s role in nAMD, as opposed to a systematic evaluation of all possible data on this topic.

Papers were included in the study if subjects of study had a confirmed diagnosis of AMD or nAMD, assessment of SD-OCT imaging parameters, and/or assessed associations between biomarkers and prognosis. The included peer-reviewed articles dated between 1990 and 2023. Non-peer-reviewed articles were not included in this study. From the literature search, a total of 148 articles were identified, of which 102 were selected per the criteria. 

This review of published literature did not require ethical approval.

## 3. Results

### 3.1. Central Retinal Thickness

CRT is calculated as the thickness of the central 1 mm of the retina from the internal limiting membrane to the RPE [11]. CRT became one of the earliest OCT biomarkers implemented as an outcome measure in clinical trials of nAMD [12]. CRT measurement has notably guided evaluation of treatment outcomes, re-treatment time, and overall clinical decisions [9].

In many large clinical trials, the BCVA improvement has been correlated with the decreases in the mean CRT, such as in the PrONTO and SUSTAIN studies [11,12,13]. Studies have also documented a relationship between the variability of CRT during anti-VEGF monotherapy and BCVA outcomes [14,15]. For example, post hoc analysis of the CATT and IVAN trials found that increased variances in retinal thickness correlated with poor visual outcomes [14,16]. In one of the first studies to focus on CRT fluctuations in patients undergoing anti-VEGF therapy, this same inverse relationship was seen when adjusted for baseline BCVA and the number of injections received [10].

However, there are several limitations to interpreting CRT as a direct biomarker of nAMD visual outcomes. The central retina comprises multiple morphologic components, such as SRF and INRF, and therefore, assessing associations from complete CRT measurements can neglect important subtleties [17]. A study that highlights this weak association between CRT and BCVA showcased an effective increase in BCVA with decreases in the CRT during the treatment loading phase, which was defined as three consecutive monthly injections followed by a 9-month maintenance period of ranibizumab [17].

This association was lost during the follow-up, as it was stated that the retinal architect was likely irreversible at this stage. Furthermore, studies which have utilized CRT as an outcome have also described its limitations due to the impact CNV has on retinal tissue and subsequent fibrosis, which can also cause both reduced BCVA and decreased CRT [12]. The use of CRT on outcome-based assessments of nAMD is therefore variable and has weak associations across different studies due to confounding factors. It is important to assess the other morphologic changes in addition to retinal thickness and their associations with functional outcomes. 

### 3.2. Retinal Fluid

#### 3.2.1. Intra-Retinal

**Figure 1:**
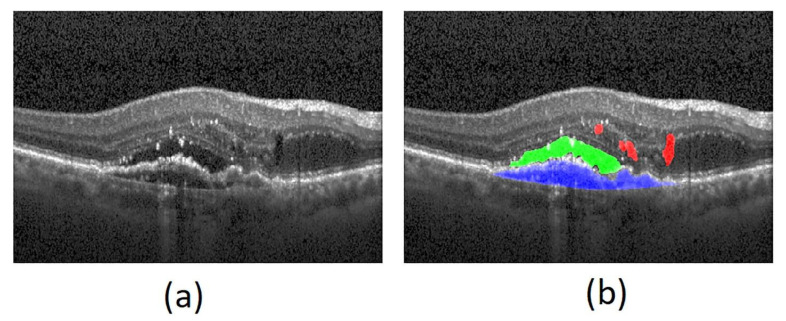
Optical coherence tomography demonstrating original (**a**) and segmented (**b**) intraretinal fluid (red), subretinal fluid (green), and sub-RPE fluid (blue). Reprinted with permission from Rashno et al. [19]. Fully automated segmentation of the fluid regions in exudative age-related macular degeneration subjects: Kernel graph cut in the neutrosophic domain. Plos One. Under Creative Commons Attribution license (https://creativecommons.org/licenses/by/4.0/ accessed on 15 January 2023).

Several retrospective studies, as well as post hoc analyses of major nAMD clinical trials, have illustrated a strong association between intraretinal fluid (IRF) and poor visual outcomes [15,18,19,20,21]. The presence of IRF itself is a key finding in nAMD pathogenesis, as it represents the leakage of fluid across an impaired blood ocular barrier due to a proliferating, invasive CNV lesion [19,22] (Figure 1). In a study of treatment-naïve patients by Jaffe et al., IRF prevalence was high with reports of IRF at baseline in 76.7% of eyes [23]. This same study by Jaffe et al. found that the IRF had a greater negative impact on VA than subretinal fluid (SRF) or sub-RPE epithelial fluid [23], pointing that this particular finding within the retina is one of the most important prognostic indicators. Many studies have found similar results, including post hoc analyses of the EXCITE, HARBOR, and ARIES trials [18,21,23,24]. 

In the Harbor study, residual IRF following treatment with ranibizumab had a significantly negative impact on vision, which increased with IRF severity. Eyes with residual, mild or moderate IRF had a mean BCVA improvement of six letters or more, compared to nine letters or more for eyes with no IRF [20,21]. Furthermore, a unique finding to this analysis involved the assessment of IRF location on outcomes. The odds of having a good VA were lower for eyes with central IRF rather than noncentral or resolved IRF [20,21], which further specifies the accuracy of this biomarker on visual outcomes.

In a study assessing OCT-derived biomarkers during ranibizumab monotherapy and combination treatment with verteporfin PDT, the strongest predictor of visual function was IRF. Patients with IRF at baseline had the lowest VA with the strongest negative predictive value for improvement throughout the study period [25]. Therefore, these findings suggest that, at baseline, a patient with IRF may have a more severe form of nAMD, which may lead to a lower potential of VA gain during monotherapy [25]. 

The presence of this biomarker has further clinical utility, as it has been observed that decisions to treat with anti-VEGF are not only based on the BCVA, but also the presence of IRF. The presence of IRF was found to be 4-times more frequent at an injection visit compared to a non-injection visit, showcasing this biomarker as a driver in clinical decision-making [26]. Additionally, the number of clinical visits during the treatment maintenance phase are noted to have a positive correlation with the absence of fluid and gain in VA [26]. The most noteworthy clinical implication of these findings is the importance IRF has on patient-specific decision making and the importance of treatment and early diagnosis in this subset population.

#### 3.2.2. Sub-Retinal

SRF is considered one of the most important OCT biomarkers in clinical practice for treatment decisions; however, its relationship with visual acuity is less clear and many studies have showcased that the presence of SRF at baseline is associated with an improved or maintained VA [21,27,28,29]. For example, analysis of the EXCITE, HARBOR, ARIES, and CATT trials all have maintained this positive relationship [18,20,21,23,24,30]. Further, in a morphological assessment of the CATT trials with a 5-year follow-up, eyes with foveal SRF had a better VA at five years than eyes that did not have SRF, and this effect was greater than at a 2-year follow-up [30]. 

Evidence from several analyses suggests that SRF is not associated with a decline in the VA and that eyes with an SRF refractory to monthly treatments of ranibizumab maintained visual improvement [27,29,31]. The FLUID trial found that participants treated with ranibizumab who tolerated SRF had a BCVA at the end of the 24 months, similar to those who received treatment aimed at eradicating all of the SRF [29]. The study importantly uncovered that with increased treatment-free intervals and tolerated SRF, patients can have equivalent visual outcomes without concern for safety [29].

CATT post hoc analyses did not find an association between the SRF and sporadic or sustained VA loss [23]. Clinically, this can have important implications, one of which includes treating patients with baseline or sustained SRF with less intense anti-VEGF treatment regimens, which potentially has several positive implications on the patient and the provider.

There are many hypotheses for the consistent association with an improved BCVA, although the underlying etiology remains unclear. Hypotheses include SRF’s role in photoreceptor protection from direct contact with disease RPE and CNV, as well as SRF influence on trophic support to the retina [30].

### 3.3. Outer-Retinal Damage

The outer retina has four layers, including the RPE, interdigitation zone (IZ), ellipsoid zone (EZ), and external limiting membrane band (ELM). Damage to any of the outer retinal layers (ORL) has been correlated with a worsening VA due to the associated photoreceptor defects [32]. Multiple types of malformations of the ORL, including outer retinal corrugations and outer retinal tubulations (ORT), can predict clinical outcomes of patients with nAMD.

**Figure 2:**
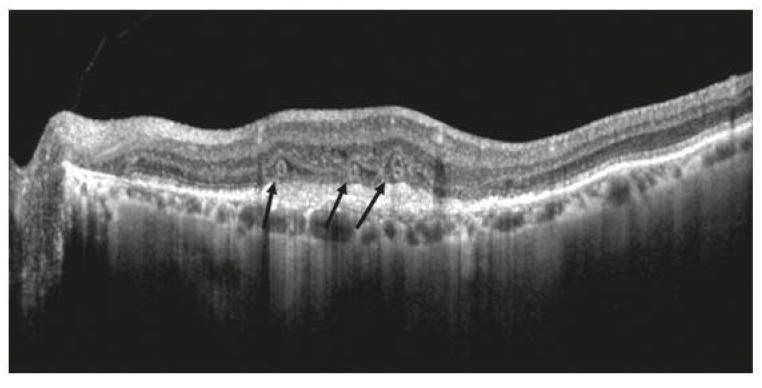
Optical coherence tomography demonstrating outer retinal tubulations in neovascular age-related macular degeneration. Tubulations in outer nuclear layer demarcated by black arrows. Reprinted with permission from Metrangolo et al. [36]. Under Creative Commons Attribution license (https://creativecommons.org/licenses/by/4.0/ accessed on 15 January 2023).

Outer retinal tubulations (ORTs) can be found in many retinal diseases, including nAMD (Figure 2). ORT describes circular, tube-like structural reconfiguration of disrupted, damaged photoreceptors [33]. They are often found in advanced nAMD, and rarely disappear with regular anti-VEGF injections. Because ORT involves the loss of photoreceptor function and is associated with geographic atrophy (GA), it is highly predictive of a worsening BCVA over time [34].

Outer retinal corrugations are groups of wavy hyperreflective materials above the Bruch membrane found on OCT imaging that are often associated with basal laminar deposits (BLamD). Outer retinal corrugations are generally limited to areas in eyes with significant CNV or GA, and are correlated with late nAMD. Outer retinal corrugations do not have a strong predictive value of visual outcomes, as they do not indicate leakage [35].

### 3.4. Hyper-Reflective Material

**Figure 3:**
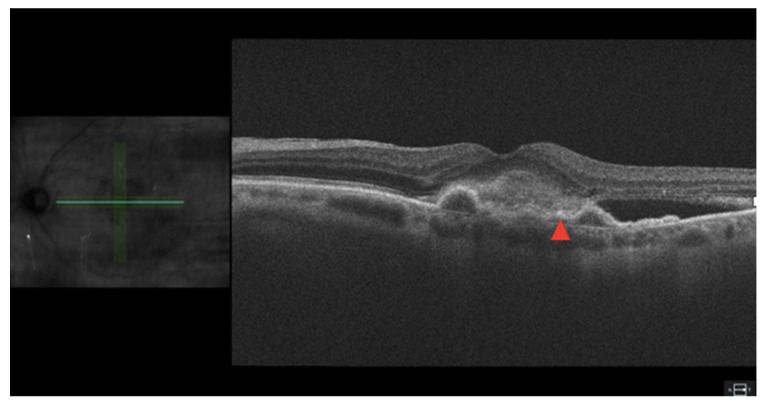
SRHM and PED in age-related macular degeneration on optical coherence tomography. Green and blue scan lines spanning location of en-face scross-section. Area of hyperreflectivity marked with a red arrow.

Subretinal hyperreflective material (SHRM) describes any hyperdense, reflective deposits in between the retina and the RPE (Figure 3). The contents of SHRM vary and can change over time, but include fibrin, choroidal neovascularization, blood, and scar tissue [37]. Because SHRM often does not completely regress with anti-VEGF treatments and can disrupt the photoreceptors of the retina, they can have maintained and progressive deleterious vision status outcomes [38]. Though they may not entirely cure patients of SHRM, anti-VEGF treatments in the eyes with nAMD have shown to improve EZ integrity, SHRM volume, and SHRM thickness, which are all highly correlated to the BCVA [39].

Some SHRM descriptors that could provide clinical utility are size, configuration, reflectivity, and structural integrity of certain retinal layers. Even though the shape of SHRMs is not associated with a difference in the BCVA, the size of SHRMs in both height and width is highly correlated with a worse BCVA, with increased width being the strongest predictor. Patients can compensate better with narrower SHRM lesions and are therefore less harmful than wider lesions, both having multiple lines of hyperreflectivity and increased reflectivity in SHRM portend a poorer prognosis [40]. SHRM also increases the risk of scar formation in eyes with nAMD, which leads to further retinal disruption and decreased visual acuity [41].

SD-OCT is a useful imaging tool in visualizing SHRM. Algorithms using a convolutional neural network (CNN) can automatically identify and quantify SHRMs, along with fluid and pigment epithelial detachment (PED) [37]. These algorithms can be useful in quickly calculating the overall risk of worsening visual outcomes due to SHRM and other biomarkers in patients with nAMD.

### 3.5. Hyperreflecive Foci (HRF) 

The HRF are well-defined hyperreflective retinal lesions with shadowing present in all retinal layers in nAMD, near the drusen edge or around the intraretinal cystoid spaces [42]. They are believed to be a strong risk factors for the progression of AMD [43]. HRF at an inner location and in a cluster distribution is associated with nAMD. The persistence of HRF after anti-VEGF treatment has been correlated with a poorer VA at baseline. Further, a decrease in the HRF has been noted in patients responsive to anti-VEGF therapy, compared to persisting HRF in non-responders, thus it serves as a good prognostic sign [44]. 

### 3.6. Pigment Epithelial Detatchment 

**Figure 4:**
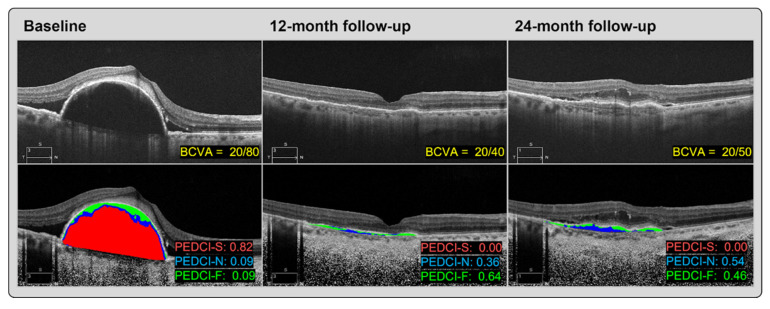
Treatment-naïve, neovascular, age-related macular degeneration with pigment epithelial detachment (PED) at baseline with a 20/80 visual acuity, at the 12-month follow-up with a 20/40 visual acuity, and at the 24-month follow-up with a 20/50 visual acuity. Top optical coherence tomography images showcase original scans, bottom scans indicate the calculated PED composition indexes. PED at baseline consists of a primarily serous composition reflected by an elevated PEDCI serous (PEDCI-S) score (colored red), whereas the 12-month follow-up scan shows a more fibrous and neovascular composition reflected by elevated PEDCI fibrous (PEDCI-F) (colored green) and PEDCI neovascular (PEDCI-N) (colored blue) scores, respectively. The recurrence of SRF in the 24-month visit corresponds to an increase in the neovascular component, PEDCI-N.

PED is the separation of the RPE from the inner collagenous layer of Bruch’s membrane (Figure 4). Large treatment trials have identified PEDs in 30–80% of nAMD patients [45]. In AMD, PEDs are classified into drusenoid, serous, fibrovascular, or mixed PEDs [46]. Drusenoid PEDs are formed by areas of soft drusen and are most frequently observed in dry AMD [47]. The pathophysiology of drusenoid PED appears to be related to the accumulation of lipids within Bruch’s membrane, leading to decreased permeability [48]. Decreased permeability then leads to the accumulation of fluids below the RPE. Serous and fibrovascular PEDs are both associated with nAMD, with some studies reporting that serous PEDs carry a more favorable long-term vision prognosis than fibrovascular PEDs [49,50]. The pathophysiology of serous PED is less understood than drusenoid PED, but may also involve the accumulation of lipids leading to impermeability of Bruch’s membrane in dry AMD or accumulation of fluid from vascularization in wet AMD [46,51,52]. Fibrovascular PED is also related to the leakage of fluids from choroidal neovascularization through Bruch’s membrane, but contains fibrovascular tissue in addition to exudation. Research has been focused on quantifying PED composition through a set of PED composition indexes (PEDCIs) to further understand prognostic factors and response to treatment in nAMD [53]. 

Cheong et al. provide a review of PED’s influence on visual acuity (VA) in nAMD [45]. Research has focused not only on VA’s association with the presence vs. absence of PED, but also on its association with morphological features, including height, width, linear diameter, area, volume, reflectivity, and content of PED. Despite a myriad of randomized control trials, as well as prospective and retrospective studies, there exists no clear consensus on the influence of any of these factors on the VA. While some studies report that the presence of PED may portend a slightly worse VA and need for more regular retreatment, other studies counterintuitively report better vision in eyes with PED. There is evidence, however, that fibrovascular PED carries an especially poor visual prognosis [54]. Hyperreflectivity, which corresponds to solid material PED, also appears to analogously associate with worsened visual outcomes [55]. Morphological parameters denoting the size of PED, including height, area, diameter, and volume, have been associated with an increased risk of an RPE tear, a serious complication of nAMD [56].

### 3.7. RPE Rips

**Figure 5:**
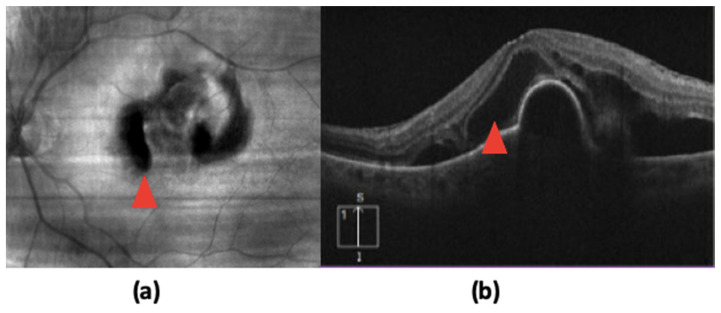
Findings of (**a**) SD-OCT scan and (**b**) retinal cross-section of an RPE rip in a patient with nAMD. Rip in two different views highlighted with red arrow.

RPE rips are a known complication of nAMD and refer to a disruption in the RPE, seen on OCT between the normal retina and border of PED (Figure 5). As the extent of RPE rip increases, the retracted RPE on OCT resembles an area with an irregular contour and hyperreflectivity with shadowing. RPE rips occur in patients with PEDs, the height being an important predictive factor [57,58]. Other important risk factors for impending RPE rips include the morphology of the PED, fibrovascular ones being at a higher risk [59], size, recent onset of less than 4.5 months [60], irregularities along the borders and interruptions in the PED in patients with nAMD on anti-VEGF therapy [61,62]. RPE rips, if present at the fovea, are associated with poor VA outcomes [63]. Visual outcomes in patients with an RPE rip are more dependent on the patient’s response to treatment rather than the underlying rip [64]. 

### 3.8. Choroidal Thickness 

The choroid is a highly vascular structure that is thickest in the subfoveal region [65]. Decreased choroidal perfusion, and subsequent thinning, secondary to vascular insufficiency, may be key to the pathogenesis of AMD [66,67]. The exact mechanism guiding decreased choroidal perfusion remains elusive, but has been demonstrated using multimodal imaging in both wet and dry AMD [68,69]. An increase in VEGF in response to vascular insufficiency may then lead to neovascularization, which results in increased choroidal thickness during active choroidal neovascularization and helps explain well-established decreases in choroidal thickness following anti-VEGF injections [70,71]. Despite this phenomenon, eyes with nAMD typically demonstrate lesser choroidal thickness than matched eyes with dry AMD, and the variability of choroidal thickness between disease states remains incompletely characterized [65,72]. The choroidal thickness is also affected by age, axial length, and other retinal pathologies [73]. 

**Figure 6:**
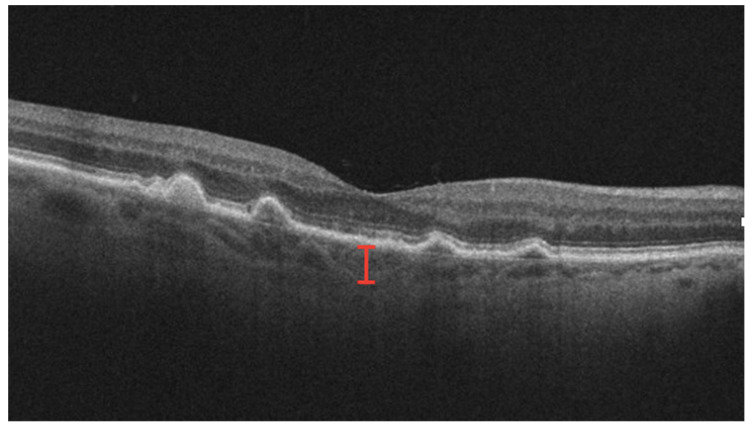
OCT with subfoveal thickness measurement demarcated by red lines spanning from retinal pigment epithelium to sclerochoroidal interface.

Significant research has focused on understanding the utility of choroidal thickness, particularly subfoveal choroidal thickness (SFCT), as a predictor of the nAMD course and prognosis (Figure 6). Studies have investigated the effects of both SFCT at baseline and changes in SFCT over disease course [74,75]. Most studies demonstrate that an increased SFCT at baseline is associated with better visual outcomes [76,77]. A thicker SFCT is hypothesized to represent preserved choroidal function and better retinal nutrition, while a thinner SFCT may denote choroidal atrophy and loss of function in chronic disease. However, existing literature does not reach a consensus on SFCT at baseline, as other studies have demonstrated an increased need for anti-VEGF treatment in patients with a thicker choroid, as well as no significant relationship between the SFCT and VA [78]. Much of the current literature on changes in choroidal thickness has focused on changes following anti-VEGF [79,80]. That anti-VEGF injections result in decreased choroidal thickness is well known, but literature is mixed as to whether the subsequent decrease in choroidal thickness is associated with an improved VA, as some studies report a significant improvement in VA, while others report no significant relationship [81,82]. Therefore, it is unclear if anatomic changes in choroidal thickness result in improved visual outcomes.

### 3.9. Choroidal Layers 

A Sattler’s layer is one of the medium-sized vessel layers, while Haller’s layer is the dilatation of the outer layer containing the large vessels [83]. Incorporating subfoveal choroidal thickness, choriocapillaris, and Sattler’s and Haller’s layer thicknesses into a linear regression model improved the coefficient of determination for the number of intravitreal injections needed after stereotactic radiotherapy [84]. It has been demonstrated that Sattler’s and Haller’s choroidal sublayers thin significantly with increasing axial eye length (and to a lesser extent with aging) in healthy eyes. In patients with a thinned Sattler’s layer in nAMD, careful OCT interpretation is needed to distinguish pathologic choroidal thinning from physiological variation of choroidal vessels. 

### 3.10. Drusen Measurements

Higher baseline drusen volume in eyes with AMD are at a higher risk for progression to nAMD or geographical atrophy [85]. Drusen length specifically, has been attributed to the risk of conversion to nAMD [86]. Quantitative SD-OCT biomarkers include the RPE–drusen complex (RPEDC) abnormal thickening and thinning (RAT). In AMD eyes, an increase in drusen and RAT volume has been noted over 2 years, and thus, these can serve as important biomarkers for the progression of AMD [87,88]. There is a 1.31 risk of progression to nAMD for every 0.1 mm^3^ increase in drusen volume [87]. The presence of reticular pseudodrusen or subretinal drusenoid deposits (SDDs) are also associated with the risk of progression to late AMD, including nAMD [89].

### 3.11. Vitreomacular Interface

Vitreomacular adhesion (VMA) and vitreomacular traction (VMT) are vitreomacular interface abnormalities seen on OCT. The presence of VMA in a patient with nAMD is said to influence the number of intravitreal injections needed for treatment. VMA is assumed to make the CNV more extensive and non-responsive to anti-VEGF treatment, thus more injections are required for treatment [90,91]. Similarly, the presence of VMT also requires more injections. The presence of VMT is associated with chronic traction and inflammation, which leads to the progression of nAMD, poor response to anti-VEGF and poorer visual outcomes. Surgical removal of the VMT can aid in the improved response of nAMD to anti-VEGF therapy [92,93]. However, they do not have any prognostic value for visual acuity [36]. 

### 3.12. Non-Exudative Lesions

In fellow eyes of patients with nAMD, there is a 6.25–27% prevalence of a non-exudative neovascular lesion with no retinal fluid [94]. These lesions are considered the precursors for the development of exudative nAMD, with a 1.21 risk of progression to exudative nAMD at 1 year [95] (Table 1).

## 4. Future Directions

Emerging technology toward enhancing the quantification of biomarkers and the optimization of reader availability, automation, and variability, have emerged. The application of artificial intelligence in OCT analysis has been shown to be a reliable and reproducible tool clinically [96,97]. Several studies have implemented deep learning and CNN to precisely quantify the biomarkers outlined in this review, including IRF, SRF, and non-fluid regions. Additionally, studies have utilized AI as a key monitoring tool to assess the progression of nAMD to an advanced disease state, as well as the progression of nAMD in the fellow eye [97]. AI is facilitating the growth of OCT imaging as a key tool in nAMD management, and it could revolutionize treatment options for patients.

OCT’s impact on clinical practice continues to evolve with AI. Most recently, the Notal Vision Home OCT (NHHO) system was developed. NVHO is a SD-OCT self-imaging device with a deep-learning-based algorithm, equipped to perform automated evaluation of images [98]. NVHO has shown to produce images with identifiable retinal fluid with increased frequency [98,99]. A prospective study of 15 patients with nAMD, by Liu et al., found that the algorithm recognized retinal fluid at an agreement rate of 83% to expert manual grading. The algorithm identified IRF better than SRF, a finding imperative to BCVA outcomes [100]. This breakthrough in OCT imaging can improve the follow-up and treatment outcomes in patients who may otherwise not be able to obtain regular in-clinic follow-up. 

An additional advancement of OCT includes the growing use of wide-field imaging systems [101,102]. Ultra-widefield imaging has become an integral component of clinical diagnosis in peripheral retinal and vascular disease. One study in nAMD observed the Clarus^TM^ wide-field imaging system had a high specificity and a 94.4% sensitivity for diagnosing nAMD [102]. The sophisticated detection of nAMD with widefield systems may aid in the early diagnosis and prompt treatment in this devastating ocular disease.

## 5. Conclusions

nAMD is one of the leading causes of irreversible blindness, and therefore, it is imperative to further understand this retinal disorder and its corresponding imaging. This review highlights thirteen OCT biomarkers that have been thoroughly assessed in the literature. On routine structural OCT imaging, caution toward pertinent findings can provide insight into potential treatment outcomes, visual function over time, and clinical decision-making. We discussed current research in utilizing these biomarkers to help prognosticate nAMD progression. Recent technologies, such as OCT angiography (OCTA), also helped to include various OCTA characteristics which can define the treatment response; however, detailed discussion on OCTA is out of the scope of the current article. 

In conclusion, retinal fluid was among the strongest indicators for both negative and positive treatment response and visual function. Other OCT biomarkers help to re-define the treatment strategies. OCT biomarkers help steer patient-specific decision-making with appropriate counseling on expectant outcomes. A more precise approach to nAMD management and anti-VEGF administration can thereby alleviate the burden of resources, time, and disease monitoring.

## Figures and Tables

**Table 1:** Summary of OCT-based biomarkers and their associated clinical significance.

Biomarker	Clinical Prognostic Association	Additional Comments
Intraretinal fluid (IRF)	Presence associated with poor visual acuity (VA) outcomes	Strong relationship
Subretinal fluid (SRF)	SRF associated with an improved VA	
Subretinal hyper-reflective material (SHRM)	Poor VA outcomes with the presence of subretinal hyper-reflective material (HRM)	Increased size and width highly correlated with worse best-corrected visual acuity (BCVA)
Outer retinal damage	Poor and worsening VA with outer retinal tubulations, but this same relationship is not seen in outer retinal corrugations	
RPE Rips	Associated with poor VA outcomes at the fovea	
Hyper-reflective foci (HRF)	HRF following anti-vascular epithelial growth factor (VEGF) treatment associated with a poor VA	
Choroidal thickness	Better VA if subfoveal choroidal thickness (SFCT) is increased at baseline	No clear consensus on relationship of thickness with VA during treatment
Central retinal thickness (CRT)	Increased CRT can lead to a decreased BCVA	Variable interpretation of CRT as predictor of outcomes
Pigment epithelial detachment (PED)	Fibrovascular PED associated with poor VA prognosis	No clear relationship among PED and VA
Choroidal Layers	Can aid in treatment regimen determination	
Drusen	Increases can predict age-related macular degeneration (AMD) progression to neovascular (nAMD)	
Vitreomacular interface abnormalities	Increased abnormalities associated with increased anti-VEGF injections	
Neovascular lesions with no fluid	If found in the fellow eye of patient with nAMD, increased risk of progression	

## Data Availability

Not applicable.
